# G-quadruplexes in H1N1 influenza genomes

**DOI:** 10.1186/s12864-021-07377-9

**Published:** 2021-01-23

**Authors:** Václav Brázda, Otília Porubiaková, Alessio Cantara, Natália Bohálová, Jan Coufal, Martin Bartas, Miroslav Fojta, Jean-Louis Mergny

**Affiliations:** 1 Institute of Biophysics of the Czech Academy of Sciences, Královopolská 135, 612 65 Brno, Czech Republic; 2 Faculty of Chemistry, Brno University of Technology, Purkyňova 118, 612 00 Brno, Czech Republic; 3 Department of Experimental Biology, Faculty of Science, Masaryk University, Kamenice 5, 62500 Brno, Czech Republic; 4 Department of Biology and Ecology/Institute of Environmental Technologies, Faculty of Science, University of Ostrava, 710 00 Ostrava, Czech Republic

**Keywords:** Influenza virus, G-quadruplex, G4Hunter

## Abstract

**Background:**

Influenza viruses are dangerous pathogens. Seventy-Seven genomes of recently emerged genotype 4 reassortant Eurasian avian-like H1N1 virus (*G4*-EA-H1N1) are currently available. We investigated the presence and variation of potential G-quadruplex forming sequences (PQS), which can serve as targets for antiviral treatment.

**Results:**

PQS were identified in all 77 genomes. The total number of PQS in *G4*-EA-H1N1 genomes was 571. Interestingly, the number of PQS per genome in individual close relative viruses varied from 4 to 12. PQS were not randomly distributed in the 8 segments of the *G4*-EA-H1N1 genome, the highest frequency of PQS being found in the NP segment (1.39 per 1000 nt), which is considered a potential target for antiviral therapy. In contrast, no PQS was found in the NS segment. Analyses of variability pointed the importance of some PQS; even if genome variation of influenza virus is extreme, the PQS with the highest G4Hunter score is the most conserved in all tested genomes. G-quadruplex formation in vitro was experimentally confirmed using spectroscopic methods.

**Conclusions:**

The results presented here hint several G-quadruplex-forming sequences in *G4*-EA-H1N1 genomes, that could provide good therapeutic targets.

## Background

Influenza viruses are deadly pathogens for humans, and more generally mammals, as well as avian species. They belong to the *Orthomyxoviridae* family and are classified into three types termed Influenza A, B and C. Among these, influenza A viruses (IAVs) pose the greatest threat to human and animal health. IAV genome is divided to 8 segments of negative-sense RNA that encodes 11 proteins [1]. Subtype classification of *G4*-EA-H1N1 is based on the antigenicity of the two major cell surface glycoproteins, hemagglutinin (HA) and neuraminidase (NA). HA protein facilitates binding of the virus to host cell receptors and subsequent endosomal fusion [2], and NA protein is responsible for binding to cellular receptors and fusion of the viral membranes, causing replication and transcription of viral RNAs in the infected host [3, 4]. The viral RNA genome (gRNA) is transcribed into mRNA and replicated through an intermediate RNA to produce a large quantity of progeny gRNA. These NAs are synthesized by the viral RNA-dependent RNA polymerase complex – polymerase basic protein 2 (PB2), polymerase basic protein 1 (PB1) and polymerase acidic protein (PA), the nucleoprotein (NP), the matrix protein (M) and the non-structural protein (NS) [5, 6].

Roots of virus H1N1 can be traced to 1918, when an avian virus overcame the species barrier to infect humans [7]. That was the beginning of a pandemic that resulted in an estimated 50 to 100 million deaths. Thereafter, influenza viruses rapidly diverged antigenically and three years later this virus was replaced by a new strain. Reassortment of influenza viruses is a major mechanism to generate progeny viruses with novel antigenic and biological characteristics [8, 9]. The emerged genotype 4 reassortant Eurasian avian-like H1N1 virus (*G4*-EA-H1N1) has become predominant in swine populations since 2016 [10] and is a new cause of concern.

Guanine quadruplexes (G4) are local nucleic acid structures formed by G-rich DNA and RNA in which four guanines fold in a planar arrangement through Hoogsteen hydrogen bonds [11, 12]. Putative quadruplex sequences (PQSs) contribute to the regulation of key biological processes [13] and have been found in the genomes of viruses (reviewed in: [14]). For example, it has been demonstrated that G-quadruplexes regulate HIV transcription and can be targeted by small compounds called G4 ligands. A comprehensive database of PQS in human all human viruses found with the Quadparser algorithm has been published [15] but these new H1N1 strains were not available at that time.

Here we analyzed 77 newly sequenced variations of H1N1 influenza virus emerged during the last years with a different algorithm, G4Hunter. There are accessible several tools to analyze PQS in genomic sequences (reviewed in [16]). We used the G4Hunter algorithm where G4 propensity is calculated depending on G richness and G/C skewness and PQS are evaluated quantitatively [17] and validated experimentally [17, 18]. We used a new G4Hunter algorithm implementation, which is suitable for batch and full genomes analyses [19, 20] and accessible as the web-tool G4Hunter web [21]. Analyses of the human genome revealed the presence of many G4-prone sequences and G4 presence has been demonstrated in a variety of species, including eukaryotes, bacteria, archaea or viruses both in silico [19, 20, 22] and confirmed experimentally [17, 23, 24]. G4 have been shown to participate in cellular and viral replication, recombination and control of gene expression [25–27]. In addition, DNA aptamers that adopt a quadruplex fold have been described as inhibitors and diagnostic tools to detect viruses [28].

In this article, we analyzed 77 *G4*-EA-H1N1 virus genomes for G-quadruplex occurrence, localization and variance to provide a rational background for PQS targeting in antiviral influenza therapy approaches.

## Results

We analyzed 616 sequences in total belonging to 77 strains of *G4*-EA-H1N1. The genome of *G4*-EA-H1N1 is 13,133 nt long and consists of 8 different segments: PB1, PB2, M, HA, NP, NS, PA and NA. PQS frequencies were analyzed according to individual *G4*-EA-H1N1 strains, and for statistical comparison we have grouped genomes according to regions of origin (10 groups based on [10]) and also according to their genomic segments (8 segments). The average GC content for the entire list of viruses is 43.37%, with minimal differences between strains, from 43.20% in the Heilongjiang strain to 43.44% in the Shandong strain. Using standard default values for the G4Hunter algorithm (window size of 25 nucleotides and G4Hunter score above 1.2), 571 PQSs were found among all genomes and all fragments. Mean PQS frequency for the whole set of sequences was 0.56 PQS per 1000 nt and PQSs cover an average of 1.58% of *G4*-EA-H1N1 genomes. The mean number of PQS per *G4*-EA-H1N1 genome was 7.42. The highest number of PQS was found in Swine Beijing 0301 2018 strain with a total of 12 PQSs, giving a PQS frequency of 0.91 PQS per 1000 nt. The lowest frequency (0.30 PQS per 1000 nt) was found in Swine Shandong S113 2014 and Swine Shandong JM78 2017 strains, where only 4 PQS with a G4Hunter score above 1.2 were found. Genomic sequence sizes, GC count, and PQS characteristics are summarized in **Table** **1**, all results for individual species and groups are in SM_02A.

**Table 1:**
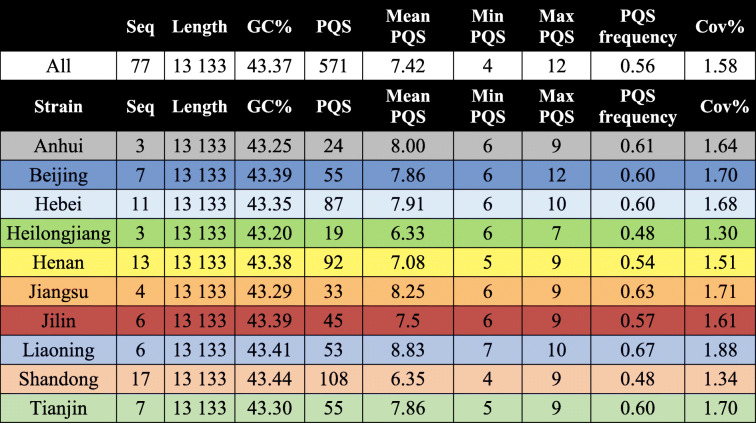
Strains of *G4*-EA-H1N1: genomic sequences sizes, PQS frequency and total counts of PQS. Seq (number of strains), Length (length of the sequence, nt), GC % (average GC content), PQS (total number of predicted PQS), Mean PQS (mean number of predicted PQS), Min PQS (lowest number of predicted PQS), Max PQS (highest number of predicted PQS), PQS frequency (PQS frequency per 1000 nt), Cov% (% of genome covered by PQS).

**Fig. 1:**
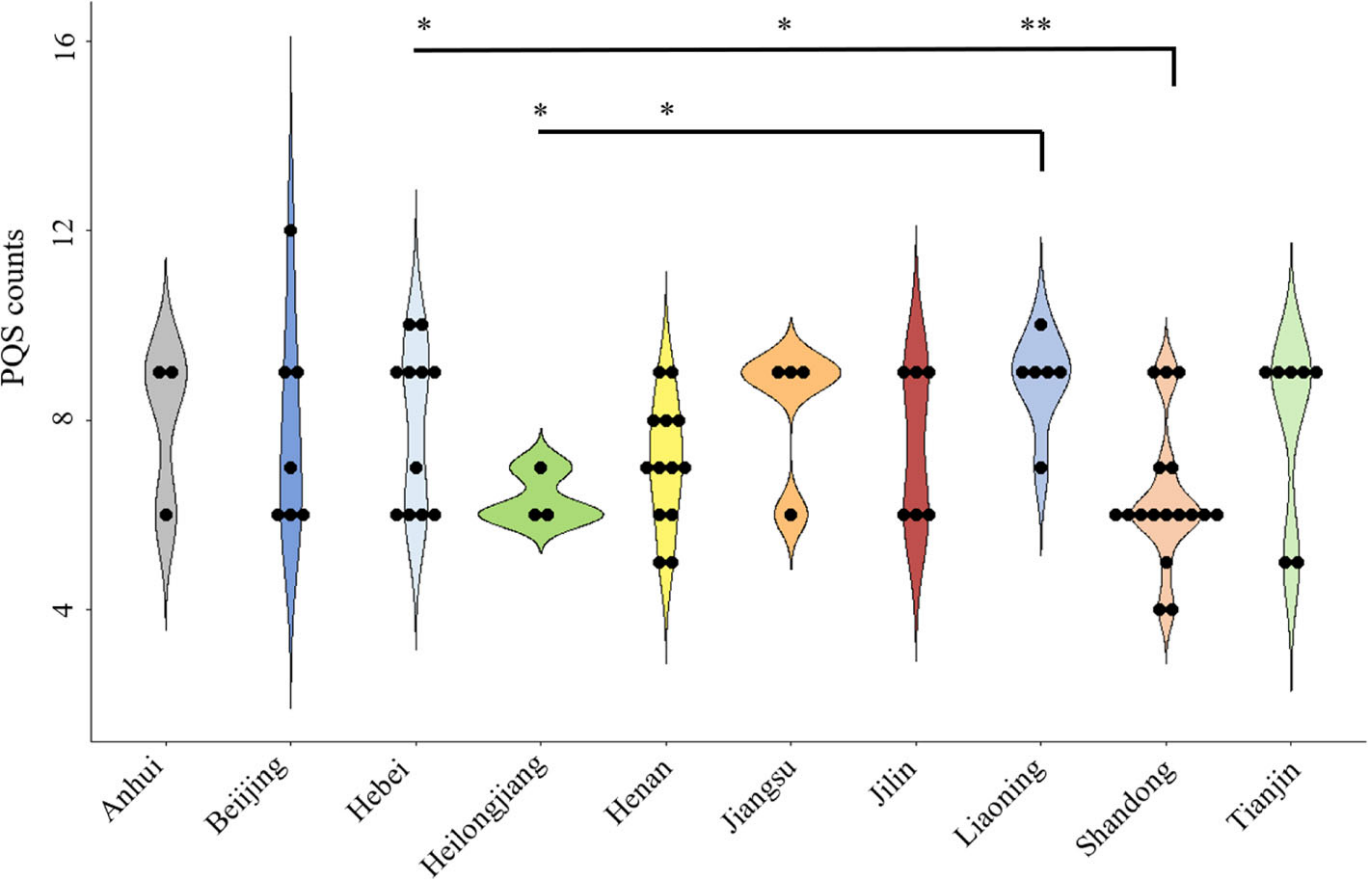
Violin plot of PQS number in *G4*-EA-H1N1 groups (SM_03). The significant differences between groups are depicted by asterisks (*p*-value < 0.05 is *; *p*-value < 0.01 is **)

Our analyses showed that PQS frequencies of *G4*-EA-H1N1 were significantly different for the Shandong group (compared to Hebei (*p* = 0.016), Jiangsu (*p* = 0.047), Liaoning (*p* = 0.0041) groups), and for the Liaoning group (compared to Henan (*p* = 0.025) and Heilongijang groups (*p* = 0,031)) (available in SM_03). Graphical representation of PQS frequencies is shown in Fig. 1**.**

We also performed PQS analyses of individual segments of influenza genomes (**Table** **2****.**); all results for segments are shown in SM_02B. Even if the global GC content in all species is very conserved, the GC content within each segment is more variable - from 41.16% in the HA segment to 47.34% in the M segment. Despite the highest CG content in HA segment, the highest mean PQS frequency was found in the NP segment (with a GC content of 46.23%), with the highest number of PQS (160). It was followed by segments NA (149 PQS) and PB2 (79 PQS). On the other hand, no PQS was found in the NS segment (which codes the non-structural protein) with a GC content of 41.52%. These data are pointing to possible functional importance of G-quadruplex in IAV genomes. All the species have 1, 2 or 4 PQS in segment NP, except for Swine Shandong LY142 2017, which does not contain any PQS with a G4Hunter score above 1.2. IAV belong to the negative-sense single-stranded RNA viruses group. Interestingly, the PQS were not distributed equally among minus gRNA which is copied for protein production (mRNA). Most of the PQSs are located in its mRNA (498 compare to 73 in gRNA). Moreover, in PB1, PB2, NP and NA segments PQS are exclusively found in mRNA (**Table** **2****)**.

**Table 2:**
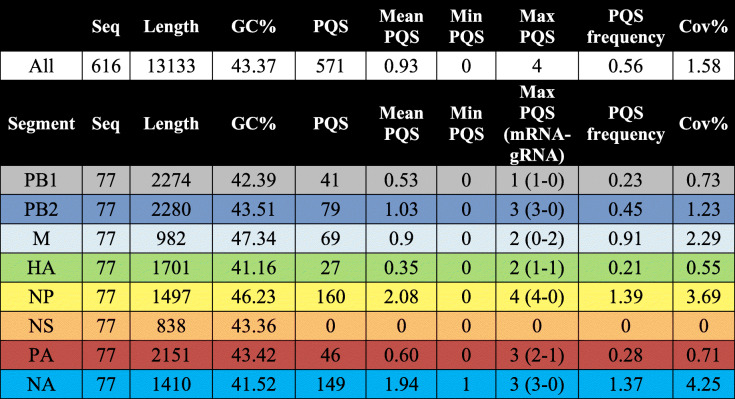
Segments of *G4*-EA-H1N1: genomic sequences sizes, PQS frequencies and total counts of PQS. Seq (total number of sequences), Length (median length of sequences), GC % (average GC content), PQS (total number of predicted PQS), Mean PQS (mean number of predicted PQS per sequence), Min PQS (lowest number of predicted PQS per sequence), Max PQS (highest number of predicted PQS per sequence), mRNA-gRNA (viral messenger RNA-viral genome RNA), PQS frequency (PQS frequency per 1000 nt), Cov% (% of genome covered by PQS).

The distribution of G4Hunter score parameters for all PQSs found in *G4*-EA-H1N1 segments is summarized in **Table** **3**. As previously found in eukaryotes, bacteria and viruses [19, 20, 22], most of the PQS have relatively low G4Hunter scores (in the 1.2–1.4 range). Only 10 / 571 motifs have a G4Hunter score above 1.4 (all in the HA segment), and no PQS was found with a G4Hunter above 1.6.

**Table 3:**
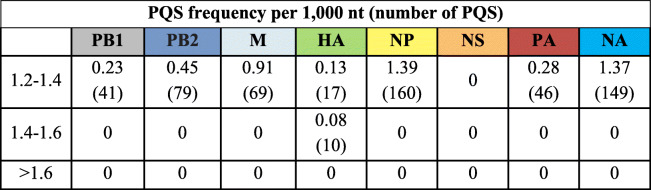
PQSs in *G4*-EA-H1N1 segments grouped by G4Hunter score (absolute values). Frequency was computed using total number of PQSs in each category divided by total length of all analyzed sequences and multiplied by 1000, the total number of PQS are in brackets.

**Fig. 2:**
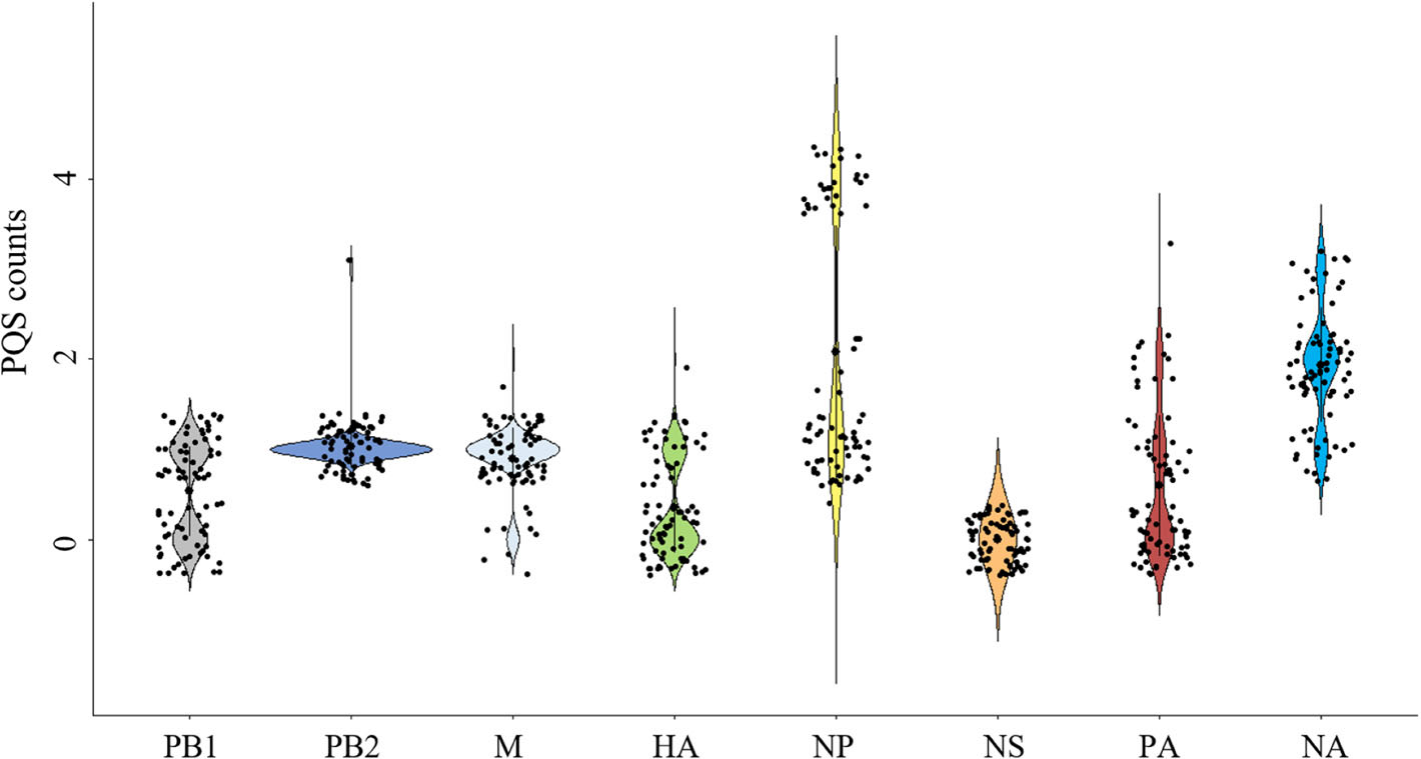
Violin plots of PQS number in *G4*-EA-H1N1 segments (SM_03). All 28 inter comparisons were significant with *p*-values < 0.05, except for PB1 vs. HA, PB1 vs. PA, and PB2 vs. M

Detailed statistical characteristics for PQS frequencies per 1000 nt, including mean, variance, and outliers, are depicted in boxplots for segments are shown in Fig. 2. Statistical evaluation of PQS in IAV segments showed the statistical differences for all comparisons except for three cases (PB1 *vs*. HA, PB1 *vs*. PA, and PB2 *vs*. M) for which differences were not significantly different.

**Fig. 3:**
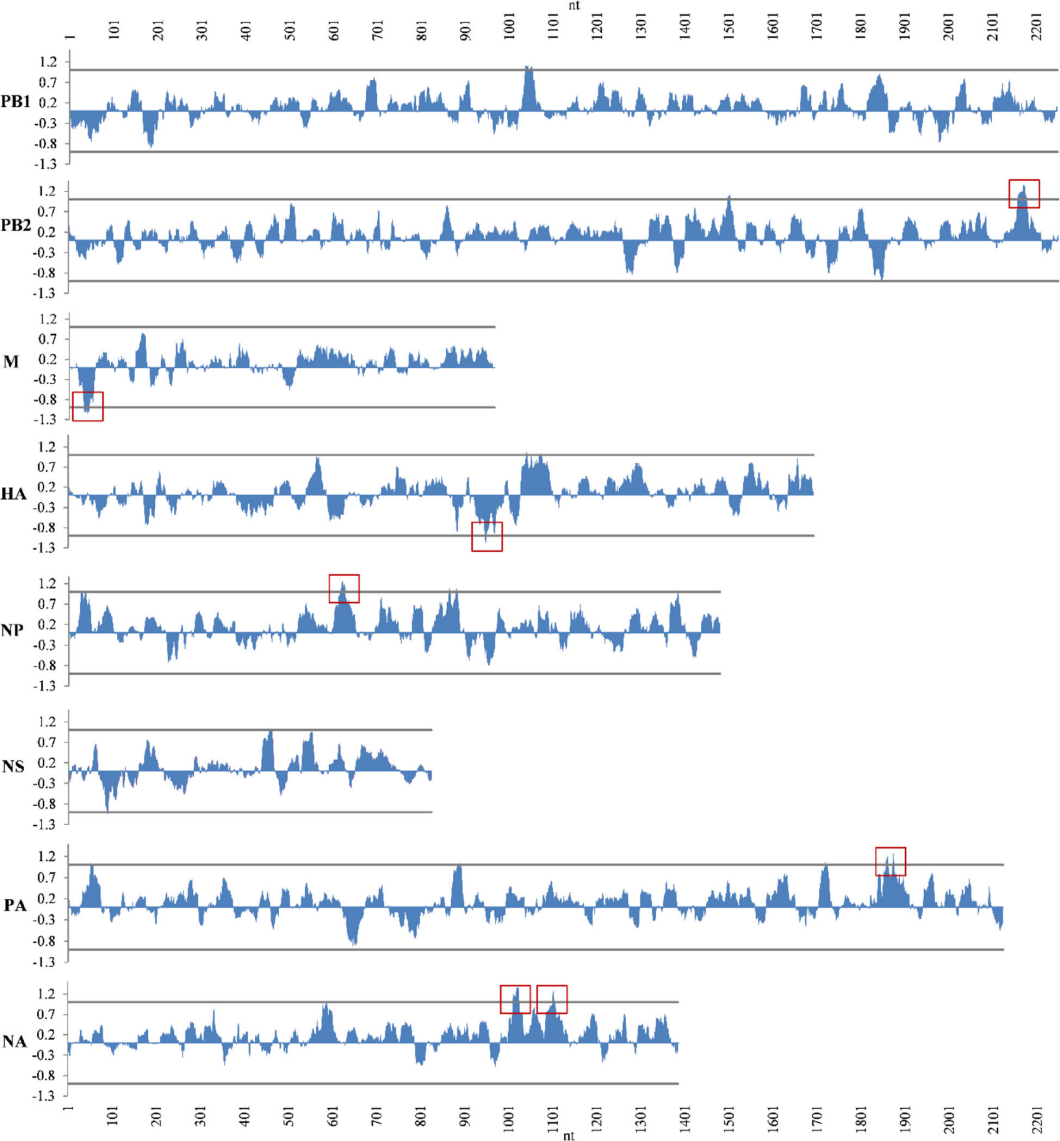
Localization of G4 prone sequences in the genome of Swine Beijing 0301 2018. Y-axis represents G4Hunter score, x-axis the length of segments. Grey lines define G4Hunter score with value of 1. PQS identified by G4Hunter with G4Hunter score over 1.2 are highlighted by red rectangles

We evaluated the localization of G4 prone sequences in the genome of Swine Beijing 0301 2018, where we found the highest number of PQS (Fig. 3**.**). From a total of 12 PQS found, 3 PQSs were in the PB2 and NA segments, 2 PQSs were located in the NP and PA segments and 1 PQS was found in the M and HA segments. The majority of PQS were found in mRNA. Ten out of all PQS were located in mRNA (with positive G4Hunter score), whereas only 2 PQS were located in negative genomic RNA (with negative G4Hunter score). Interestingly, in segment M, one PQS was located at the 3′ end of intron in negative-sense genomic RNA, near the splicing site of mRNA, which encodes M2 protein. M segment codes 2 matrix proteins – M1, which is coded by whole segment and spliced protein M2 [29]. All 10 conserved PQSs located in positive-sense RNA completely span coding regions; this is hardly surprising, as the vast majority of RNA segments are protein coding, except for short 3′ and 5′ UTRs.

**Fig. 4:**
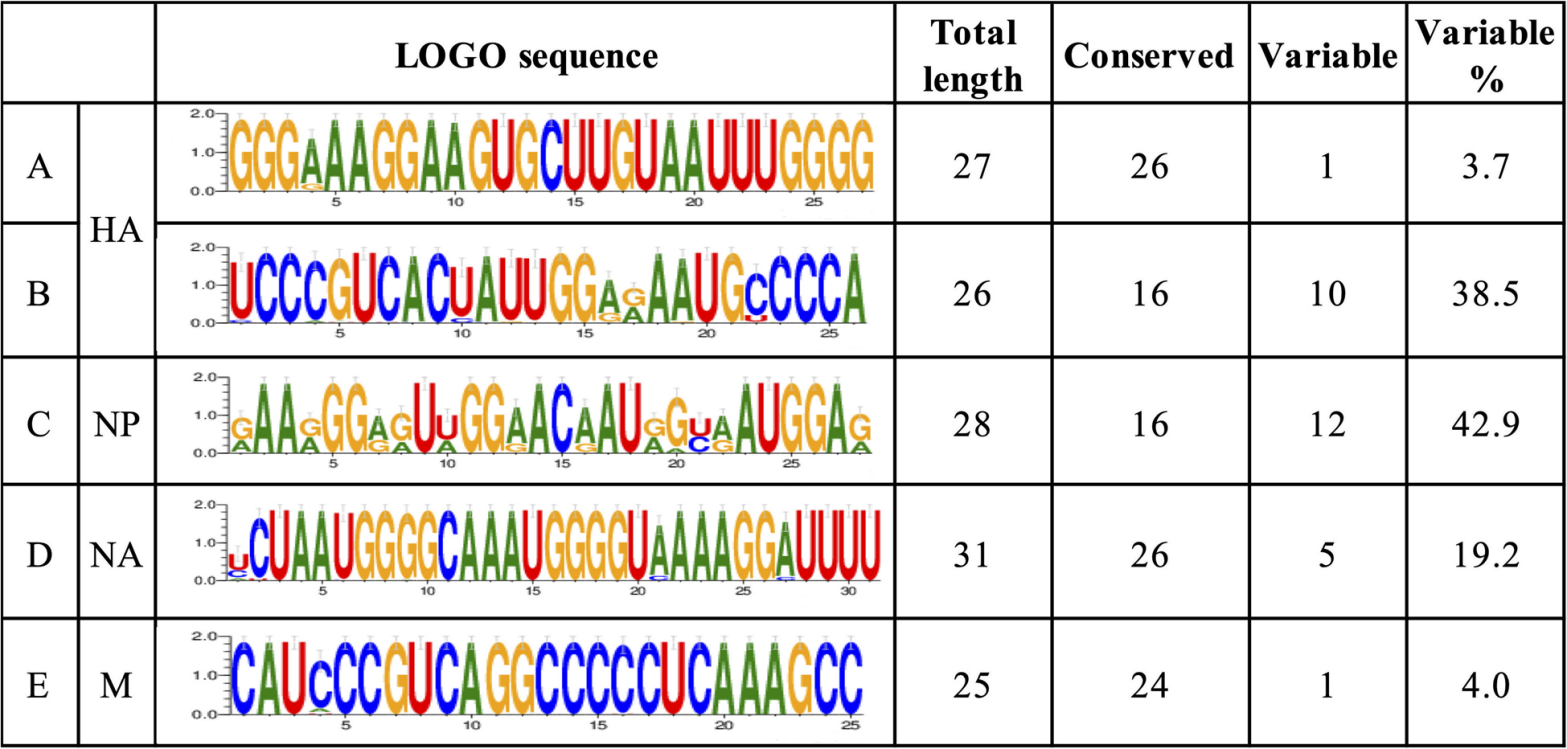
Examples of PQS motifs and their variation presented as LOGO sequences **a**. PQS with the highest G4Hunter score (1.4), **b**. PQS with the lowest G4Hunter score (− 1.2; a negative score indicates that the G-rich motif is located in negative gRNA), **c**. PQS with the most variable sequence (G4Hunter score 1.2) from NP segment, **d**. PQS with conserved GGGG-tracks (1.1) and **e**. PQS conserved sequence (− 1.2,). Perfectly conserved nucleotides are represented by full size letters. All sequence logos are shown in SM_04

A comparison of genomes revealed that some, but not all, PQS motifs were highly conserved. We align all predicted PQS and generate their LOGO representation (SM_04). Selected LOGO sequences with the highest positive and negative G4Hunter scores and with the most variable nucleotides are shown in Fig. 4**.** For example, in the M and HA segments, we found PQS in which only 1 nucleotide (out of 25 and 27, respectively) is variable within the PQS motif among all 77 strains. In contrast, other PQS sequences were poorly conserved / extremely variable (for example, the PQS sequence “**C**” in the NP segment has 12 / 26 variable nucleotides in its PQS; this can lead to significant variations in G4Hunter score and quadruplex propensity).

Overall, *G4*-EA-H1N1 genomic sequences are very variable. The analyses of 77 *G4*-EA-H1N1 genomes show a global variation of 23.4%. Therefore, the high sequence conservation of some PQS (two of them have a variation < 4.0% in Fig. 4) suggests they play crucial roles in influenza virus. The PQS sequence with the highest G4Hunter score is also the most conserved among all found PQS. Similarly, another sequence with two GGGG runs (Fig. 4d), which could form bimolecular G4, has 100% conservation within the G-tracts.

**Fig. 5:**
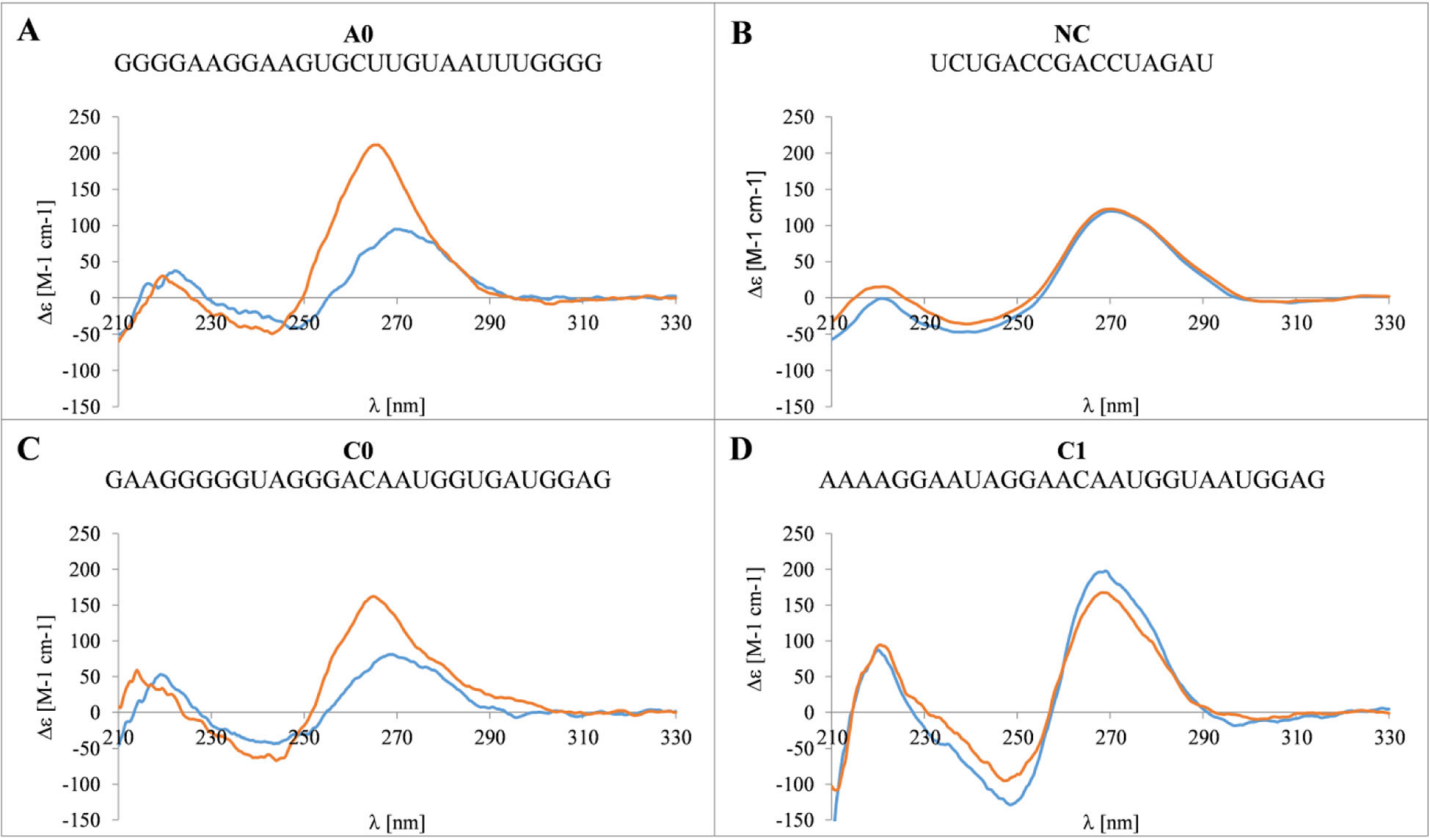
Circular dichroism (CD) spectra of selected PQS in 1 mM sodium phosphate buffer (pH 7) (blue lines) or in 1 mM sodium phosphate (pH 7), 10 mM potassium phosphate (pH 7), 90 mM KCl buffer (orange lines); **a**. Oligonucleotide AO **b**. Negative control (NC); **c** Oligonucleotide CO; **d** Oligonucleotide C1

We then determined if the quadruplex-prone sequences identified in silico actually form G4 in vitro. This experimental confirmation is important for these motifs, as their G4-Hunter scores are relatively low, and some candidate sequences may prefer formation of other structures and/or fail to form stable G4 (100% confidence in predicted motifs can only be achieved for relatively high scores, typically above 1.6). To confirm the ability of the most conserved PQS to form G4 in vitro, we used a combination of two biophysical methods, circular dichroism (CD) spectroscopy and the Thioflavin T (ThT) fluorescent assay [30, 31], results are shown in SM_06. We tested nine synthetic oligonucleotides derived from the LOGO sequence listed in Fig. 4. For sequences A, C and E we analyzed two variants, one with the highest and one with the lowest possible G4Hunter score. Quadruplex formation was confirmed for 5 out of 8 analyzed sequences (**Table** **4**). G-quadruplex formation in vitro was confirmed by CD spectroscopy as the shift of the peak from 270 to 264 nm and a stronger signal in the presence of K^+^ ions (potassium ions stabilize the G4 structure). An example of positive result is presented in Fig. 5**, part A** for a conserved sequence derived from HA fragment and in Fig. 5**, part C** for the sequence from NP fragment with the highest possible G4Hunter score. An example of negative result acquired by CD spectroscopy is shown in Fig. 5**, part B** for a negative control sequence with the G4Hunter score of 0.37 and in Fig. 5**, part D** for the sequence derived from the NP fragment with the lowest possible G4Hunter score.

**Table 4:**
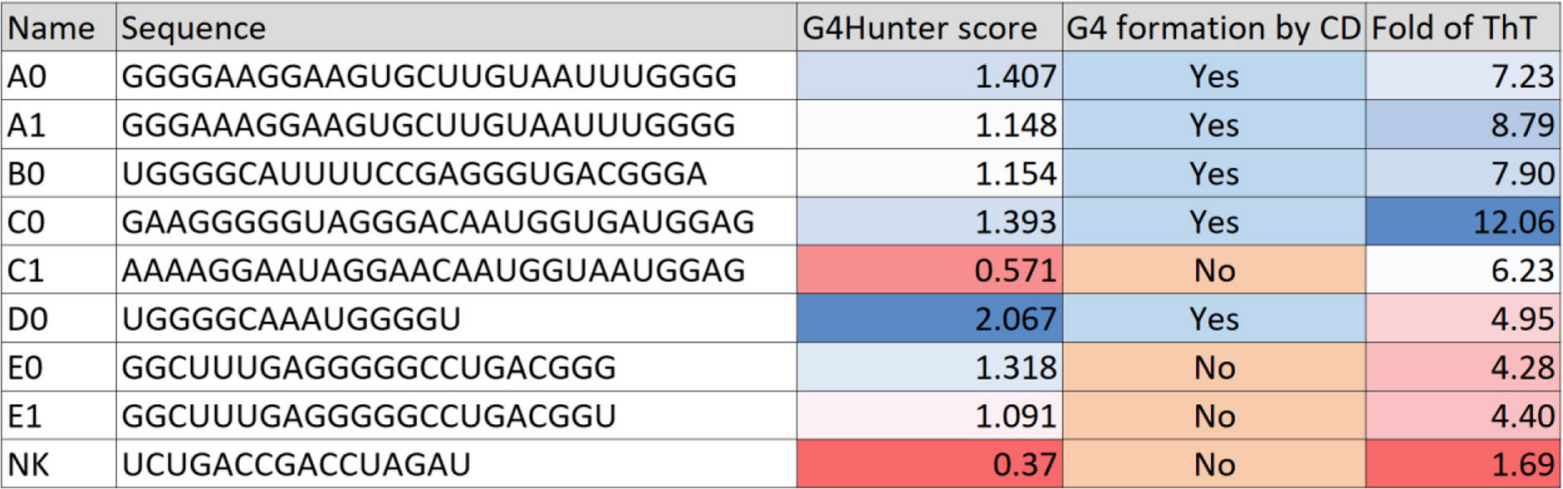
Summary of the in vitro G4 formation analyses by CD spectroscopy and ThT fluorescent assay in vitro. Sequences are shown in the 5′ to 3′ direction; all oligonucleotides are RNA. For G4 formation by CD, “Yes” indicates that a CD signature typical of a parallel G4 structure in the presence of K^+^. The result of CD spectroscopy was considered positive in the case of a blue-shift of the positive ellipticity peak (from 270 to 264 nm) and a stronger signal in the presence of K^+^ ions. Ratio between ThT fluorescence in the presence of oligonucleotide and background fluorescence of ThT alone is presented in the last column. The light-up effect ((“Fold of ThT”) refers to fold increase in Thioflavin T fluorescence emission when the candidate sequence is added: the higher this increase, the more likely is the structure to form a G4 motif.

## Discussion and conclusions

The influenza viruses pose a global public health concern. Influenza claims 250,000–500,000 lives annually, even though vaccines and antiviral drugs are available. There is therefore an urgent need to develop antiviral drugs with novel mechanisms of action. Noncanonical nucleic acid structures play an important role in basic biological processes [32] and it has been shown that G4s may be used as targets for therapy [33, 34]. Therefore, noncanonical structures in the H1N1 viral genome could serve as possibly targets for antiviral therapy. In this study, we provide a detailed analysis of PQSs occurrences, frequencies and distributions in the contemporary emerged *G4*-EA-H1N1 strains.

We found a total number of 571 PQS in all 77 *G4*-EA-H1N1 genomes. Interestingly, the number of PQS in close *G4*-EA-H1N1 relatives varied from 4 to 12. Analyses of variability pointed to the importance of some PQS: even if genome variation of influenza virus is extreme, the PQS with the highest G4Hunter score is nearly perfectly conserved in all tested genomes. Comparison of segments shows significant differences among individual *G4*-EA-H1N1 segments. While the highest mean PQS frequency was found in the NP segment (1.39), which codes for a protein playing a central role in viral replication [35], the most abundant viral protein in infected cells [36, 37] and the most promising drug target [37] – no PQS was found in the NS segment (which codes for the non-structural NS protein).

To evaluate the presence of the PQS in individual fragments we randomize five-times the RNA sequences of the Liaoming group (the group with highest PQS frequency) and as well in the Shandong group (the group with the lowest PQS frequency). A significant difference in PQS frequency was found between reference and randomized sequences for the NA segment of both groups (SM_05). On the other hand, the PQS frequency was not significantly different for other frogments, except for a depletion in the PA fragment in the Shandong group. These results are in agreement with recently proposed hypotheses that viruses causing acute infection are depleted in PQS (Bohálová N, Cantara A, Bartas M, Kaura P, Šťastný J, Pečinka P, Fojta M., Mergny J-L., Brázda V.: Analyses of viral genomes for G-quadruplex forming sequences reveal their correlation with the type of infection (submitted). A similar finding was published for SARS-CoV-2 [22]. On the other hand, the abundance of PQS in in the NA segment suggests its important evolutionarily conserved function.

Of note, none of the PQS identified here match a classical quadruplex consensus, in which four runs of three or more guanines are separated by 7 nucleotides or less, as predicted by Quadparser [38] with default parameters. As RNA G4 structures tend to be more stable than DNA, some of the motifs found here are still likely to form quadruplexes under physiological conditions, and this was experimentally confirmed using a combination of two biophysical methods. Given that all G4Hunter scores were relatively low, G4 formation was not a given, and needed the experimental confirmation. Our results show that the most conserved PQS in HA fragment, one with a conserved G run in NA fragment, as well as some others are capable to form G-quadruplex structure in vitro, as shown by CD spectroscopy and by Fluorescence light-up measurements, while two sequence with low G4Hunter scores (< 0.6) did not form stable quadruplexes at room temperature. Interestingly, and as observed previously, the “grey zone” for which a sequence may well form a quadruplex or not seems to be centered around 1.1–1.2, and we have several sequences with relatively similar scores (between 1.09 and 1.31) which give different outcomes. G4Hunter is therefore not perfect – as all current prediction tools – and we are currently working on modifying parameters to improve accuracy. This may prove more difficult for RNA than for DNA, as we currently have access to far more experimental data on DNA than on RNA oligonucleotides.

In contrast to Quadparser, G4Hunter does not pick individual G-tracts to propose a core quadruplex with three loops. As demonstrated by a number of studies, the universe of G4-forming sequences is very diverse, and may involve bulges or snapback motifs, allowing individual, isolated guanines to participate in G-quadruplex formation. There are, of course, specific cases in which G participating to G-quartets can reasonably be assumed. For example, a bimolecular four-layer G4 motif can be predicted within the D motif for the GGGGCAAAUGGGG region. In addition, for all motifs, one can imagine an intramolecular structure, provided that *i)* two-layered RNA G-quadruplexes are stable, and that *ii)* zero-nucleotide loops are allowed (such propeller loops have been found in a limited number of cases). In addition, one cannot exclude that isolated G also contribute to the core quartets. For this reason, in the absence of high resolution structures, it is rather premature to propose which G within these motifs are involved in G4 formation. These observations further illustrate that it is not possible to cover all G4-forming motifs with a single general consensus sequence.

These structures may offer opportunities for regulation and targeting by G4 ligands. Interestingly, several conserved PQS contain two GGGG runs, which may allow stable bimolecular G4 formation, as suggested for genome organization in other viruses including SARS-CoV-2 [39, 40].

Both strands, negative-sense genomic RNA and positive-sense mRNA, were analyzed for the presence and distribution of PQS as both RNAs are involved in lifecycle of the virus. Our result show that PQS are not evenly distributed but are mostly located on the RNA positive strand, thereby they may be involved in translation and splicing regulation. The genome of IAV is not stable and varies remarkably among strains [10, 41]. Comparison of PQS in various strains demonstrated that several PQS in the M segment and HA segment are highly conserved and therefore may be considered as suitable candidate targets with therapeutic potential. The HA segment (hemagglutinin) codes for a primary viral protein, which is recognized by the immune system and also is the primary target for vaccine design [42]. HA contains two subunits: HA1, which is responsible for receptor binding and HA2, which function is to support HA1 and mediates membrane fusion during viral entry [43]. Moreover, the conserved PQS in the HA sequence has the highest G4Hunter score among all found PQS. Another highly conserved PQS (just 1 variable nucleotide as shown in Table 3) was found at the 3′ end of an intron in negative-sense genomic RNA, near the splicing site of M2 protein. M1 is the only viral structural component which plays a major role in virus particle assembly [44]. M2 is a transmembrane ion channel protein which plays an important role in early stages of viral entry. Moreover, the M segment of 2009 H1N1 pandemic influenza virus was derived from the Eurasian avian-like swine lineage and was shown to affect neuraminidase activity and therefore might also have a potent effect on transmissibility [45].

Compared to strong depletion of PQS in the contemporarily outbreaking SARS-CoV-2 virus [22, 46], the genomes of IAV contain a highly conserved PQS, which could serve as a selective target. Our comprehensive analyses confirmed that several candidates adopt a quadruplex fold, arguing for the therapeutic potential of these PQS as targets for specific ligands.

## Methods

### Source of DNA sequences

The complete set of 616 sequences of 77 *G4*-EA-H1N1 genomes (each genome is divided into 8 segments) was downloaded on July 3, 2020 from the Genome database of the National center for Biotechnology Information (NCBI) [47]. NCBI accessions are shown in SM_01.

### Process of analysis

All sequences belonging to 77 strains of *G4*-EA-H1N1 were analyzed with the G4Hunter Web tool [21], which is capable to read the NCBI identifier of the sequences uploaded in a .csv files. Default parameters for G4Hunter were set to “25” for window size and 1.2 or above for G4Hunter threshold score. G4Hunter score was grouped to the five intervals: 1.2–1.4, 1.4–1.6, 1.6–1.8, 1.8–2.0 and 2.0 and more, as previously performed for the Bacteria domain [19]. PQS frequencies were analyzed according to individual *G4*-EA-H1N1 strains, grouped according to regions of isolation and according to their eight genomic segments. All results including information about the size of genomic DNA sequence, number of PQS and statistical data are shown (SM_02A – grouped by region and SM_02B – grouped by segments).

### Statistical evaluation

Statistical analysis of normality was made with the Shapiro-Wilk test. Since it was found that the data do not have a normal distribution, we used Kruskal-Wallis signed rank test to evaluate significant differences among strains and segments. Post-hoc multiple pairwise comparison by Dunn’s test with Bonferroni correction of the significance level was applied with *p*-value cut-off 0.05. Data are available in SM_03.

The statistical significance analysis of the found PQS with respect to the same genome composition in a scrambled order was performed. The strains with the highest and lowest PQS frequency were selected. Reference sequences of individual segments of selected strains were five times randomized by the program Sequence Manipulation Suite – Shuffle DNA [48]. PQS frequencies were evaluated equally in reference and randomized sequences and plotted (SM_05).

### Construction of LOGO sequences

NCBI sequences by list from SM_01 in FASTA format were downloaded and the dataset was uploaded to SnapGene (Align Multiple DNA Sequences) program. For every PQS we used the corresponding sequences from all *G4*-EA-H1N1 genomes and alignments by Clustal Omega tool [49] were generated. All found PQS were searched in aligned sequences and WebLogo 3 [50] was used for generating LOGO sequences, all predicted PQS and LOGO sequences for PQS are available in SM_04.

### CD spectroscopy

Synthetic oligonucleotides were purchased from Sigma-Aldrich and diluted in water. Oligonucleotides were heated at 85 °C for 3 min in 1 mM sodium phosphate buffer pH 7 or 1 mM sodium phosphate pH 7, 10 mM potassium phosphate pH 7 and 90 mM KCl buffer and slowly cooled down to room temperature. CD measurements were carried out with a Jasco 815 (Jasco International Co., Ltd.,Tokyo, Japan) dichrograph in 1 cm path-length microcells at 23 °C. A set of four scans with a data pitch of 0.5 nm and 200 nm/min scan speed was averaged for each sample. CD signal is expressed as the difference in molar absorption, Δε, of the left- and right-handed circularly polarized light [31].

### ThT fluorescent assay

Synthetic oligonucleotides were purchased from Sigma Aldrich and diluted in water. Oligonucleotides were diluted to 2 μM final concentration in 100 mM Tris-HCl pH 7.5 and 100 mM KCl buffer, heated at 85 °C for 3 min and slowly cooled down to room temperature. ThT was diluted in water to 1 μM final concentration and 100 mM KCl was added. Experiments were performed in a 384-well microplate from CORNING (Flat Bottom Black Polyester). Each condition was tested in triplicate. Measurements were performed at room temperature. Oligonucleotides and ThT were mixed at 0.5:1 M ratio to a final volume of 20 μl. Fluorescent emission was collected at 490 nm after excitation at 425 nm with a microplate reader (Spark, Tecan) [30].

## Data Availability

Data availability: all data are enclosed in the Supplementary materials (SM). SM_01: NCBI accession numbers for all tested sequences. SM_02: G4Hunter analyses results (A) strains (B) fragments. SM_03: Statistical evaluation. SM_04: PQS LOGO representation. SM_05: Statistical analysis of randomized sequences (A) Shandong (B) Liaoning. SM_06: Circular dichroism (CD) spectroscopy and the Thioflavin T (ThT) fluorescent assay results.

## Abbreviations

G4-EA-H1N1: Genotype 4 reassortant Eurasian avian-like H1N1 virus
PQS: Potential quadruplex-forming sequence
G4: Quadruplex
g-RNA: Viral RNA genome
IAV: Influenza A virus
HA: Hemagglutinin
NA: Neuraminidase
PB1: Polymerase basic protein 1
PB2: Polymerase basic protein 2
PA: Polymerase acidic protein
NP: Neuroprotein
M: The matrix protein
NS: The non-structural protein
CD: Circular dichroism
ThT: Thioflavin T
